# Dihydroergotamine and triptan use to treat migraine during pregnancy and the risk of adverse pregnancy outcomes

**DOI:** 10.1038/s41598-021-97092-y

**Published:** 2021-09-29

**Authors:** Anick Bérard, Shannon Strom, Jin-Ping Zhao, Shashi Kori, Detlef Albrecht

**Affiliations:** 1 Faculty of Pharmacy, University of Montreal, Montreal, QC H3T 1J4 Canada; 2 Research Center, CHU Sainte-Justine, Montreal, QC H3T 1C5 Canada; 3 Faculty of Medicine, Université Claude Bernard, Lyon 1, 69622 Lyon, France; 4 Satsuma Pharmaceuticals, Inc, San Francisco, CA 94080 USA

**Keywords:** Epidemiology, Drug development, Outcomes research, Paediatric research

## Abstract

Migraine is prevalent during pregnancy. Antimigraine medications such as dihydroergotamine (DHE) and triptans have been associated with adverse pregnancy outcomes in individual studies but lack of consensus remains. We compared the risk of prematurity, low birth weight (LBW), major congenital malformations (MCM), and spontaneous abortions (SA) associated with gestational use of DHE or triptans. Three cohort and one nested-case–control analyses were conducted within the Quebec Pregnancy Cohort to assess the risk of prematurity, LBW, MCM, and SA. Exposure was defined dichotomously as use of DHE or triptan during pregnancy. Generalized estimation equations were built to quantify the associations, adjusting for potential confounders. 233,900 eligible pregnancies were included in the analyses on prematurity, LBW, and MCM; 29,104 cases of SA were identified. Seventy-eight subjects (0.03%) were exposed to DHE and 526 (0.22%) to triptans. Adjusting for potential confounders, DHE and triptans were associated with increased risks of prematurity, LBW, MCM, and SA but not all estimates were statistically significant. DHE was associated with the risk of prematurity (aRR: 4.12, 95% CI 1.21–13.99); triptans were associated with the risk of SA (aOR: 1.63, 95% CI 1.34–1.98). After considering maternal migraine, all antimigraine specific medications increased the risk of some adverse pregnancy outcomes, but estimates were unstable.

## Introduction

Migraine is a common neurovascular disorder with a 1-year prevalence of 9–22% in women and a peak prevalence during the reproductive years^1^. Improvement of migraine severity has been reported in 55–90% of women during pregnancy^2^. However, many women continue to suffer from migraine attacks during pregnancy and seven percent (7%) have a first-onset migraine during pregnancy^2^.

There is little data on the use of therapies for the acute treatment of migraine during pregnancy. Therapies for the acute treatment of migraine are categorized into migraine specific and migraine non-specific drugs. Specific drugs include ergot alkaloids (dihydroergotamine (DHE)) and selective serotonin 5-HT receptor agonists (triptans). Studies have reported that up to 70% of pregnant women with migraine use antimigraine drugs^3,4^.

The antimigraine activity of DHE mesylate is likely related to its agonist activity at 5-HT_1B_, 5-HT_1D_, and 5-HT_1F_ receptors leading to meningeal vasoconstriction and trigeminal inhibition^5,6^. Despite being commercially available since the mid-1940s, there is limited data on the safety of DHE in human pregnancy, likely because DHE is not recommended during pregnancy as a result of potential teratogenic effects reported in reproductive toxicology studies in rats and rabbits undertaken in the course of developing a liquid nasal spray formulation during the 1980s and 1990s. Ergot alkaloids, such as DHE, have a contractile effect on uterine smooth muscle and may cause decreased uterine blood flow^7^. Reinterpretation of reproductive toxicology study results and the established uterotonic and vasoconstrictive effects of DHE suggest any potential risk of adverse pregnancy outcome associated with DHE use could depend upon when in the course of pregnancy exposure occurs. Moreover, a retrospective analysis of DHE exposures among subjects within a large human pregnancy cohort found that DHE exposure did not statistically significantly increase the risk of low birth weight (LBW), major congenital malformations (MCM), or spontaneous abortions (SA); however, DHE exposure was associated with a four-fold increased risk of prematurity^8^.

Triptans are effective for the acute management of migraines and exert their effects by binding to the serotonin 5-HT receptors, thereby leading to vasoconstriction and inhibition of neuronal inflammation^9^. Even though the first triptan, sumatriptan, has been on the market for more than 20 years, data on the safety of triptans in human pregnancy is limited. Based on the accumulated evidence from the sumatriptan pregnancy registry^3^ and other studies or meta-analyses^10–14^, the risk of MCM was reported to be similar to the baseline risk in the general population (3–5%)^9^. Bérard and Kori^8^ however reported an increased risk of SA associated with the use of triptans (any types combined) during pregnancy. A meta-analysis showed that gestational use of triptans was not associated with the risk of MCM but did suggest an increased risk of SA^15^.

Because migraine attacks are common during pregnancy, and guidelines are changing rapidly, more data are needed to guide prescribers and help better characterize the relative benefits and potential risks, to both women and their fetuses, of therapies utilized for the acute treatment of migraine. Analyses should ideally take into account that migraine itself may be associated with an increased risk of prematurity, LBW, and SA as several studies^2,16–18^ have shown. At present, hardly any studies looked specifically at DHE in large cohorts of pregnant women over long periods of time; or compared DHE to triptans, which are both migraine medications, within a single population-based pregnancy cohort. In addition, fewer have studied all clinically relevant adverse pregnancy outcomes together while accounting for important potential confounders. In addition, although Bérard and Kori^8^ studied DHE and triptan users in pregnancy, they did not fully take into consideration indication bias or potential bias due to unmeasured confounders.

Hence, the aim of this study was to quantify the risk of prematurity, LBW, MCM, and SA associated with gestational use of DHE, and compare DHE and triptan use during pregnancy in terms of adverse pregnancy outcomes, within the population-based Quebec Pregnancy Cohort (1998–2015).

## Results

**Figure 1:**
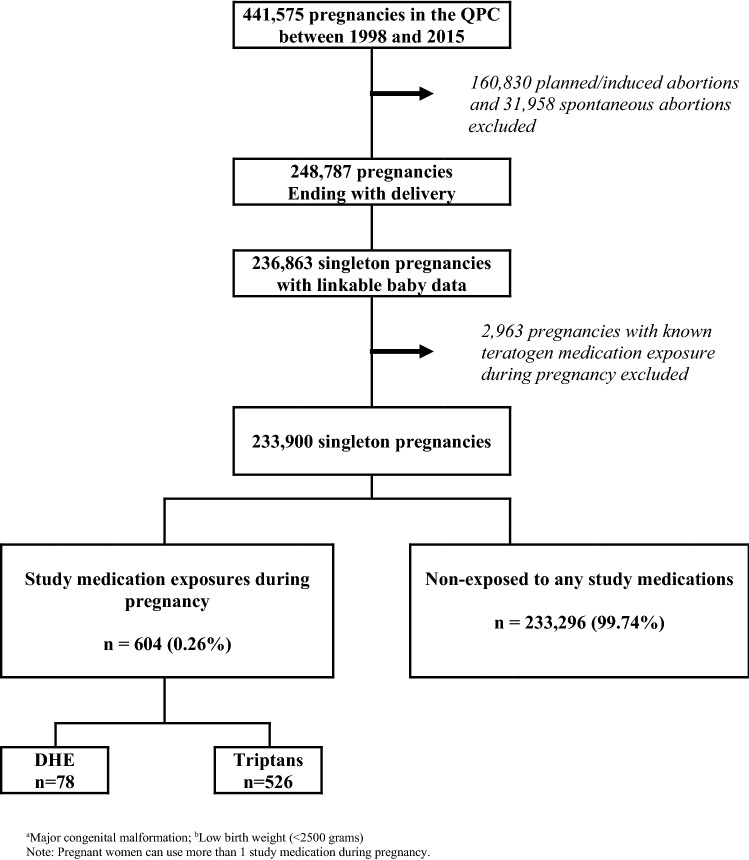
Selection of the study population for analyses on MCM^a^, prematurity, and LBW^b^.

**Figure 2:**
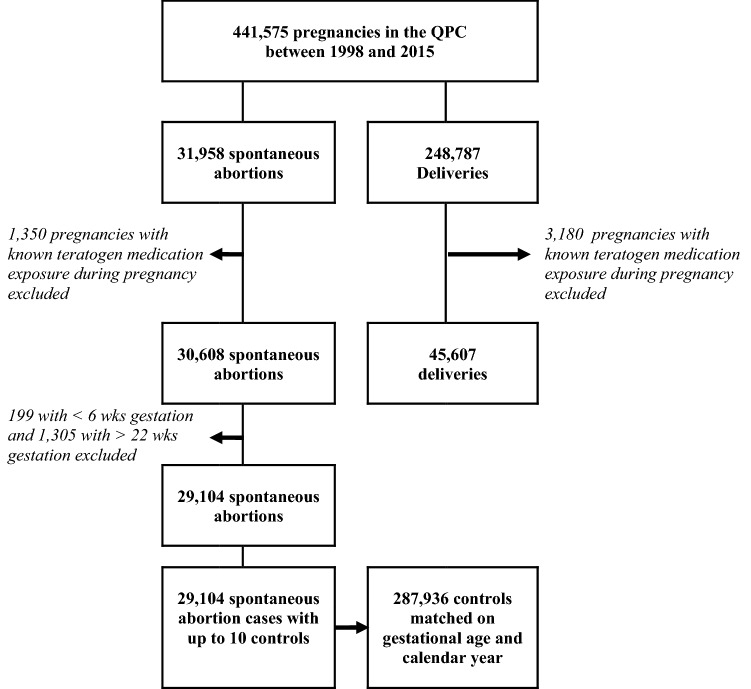
Selection of cases and controls for the analyses on spontaneous abortion.

Of the 441,575 pregnancies included in the QPC between 1998 and 2015, 233,900 met inclusion criteria and were considered for the analyses on prematurity, LBW, and MCM; 78 (0.03%) were exposed to DHE, and 526 (0.22%) to triptans (Fig. 1). For analyses on SA, 29,104 cases met our study definition, and 287,936 controls were sampled and matched on gestational age and calendar year of the event (Fig. 2).

## Prematurity

In this study, 15,688 (6.7%) pregnant women had a premature delivery (Table 1); deliveries were mostly between 32 and 36 weeks’ gestation (Table 2). Those with a preterm delivery were older; less likely to be on welfare; were hypertensive or diabetics; more likely to be smokers or be using illicit drugs; had more hospitalizations, physician visits and use medications other than the ones studied here; they were also more likely to be followed by an obstetrician and take high dose folic acid (Table 1). Of the 78 DHE exposures during pregnancy, all of them were before the 15th weeks of gestation; of the 526 triptan exposures, all were before the 21st week of gestation. Adjusting for all potential confounding variables including maternal migraine, DHE use during pregnancy was associated with an increased risk of prematurity (aRR, 4.12, 95% CI (1.21, 13.99); 19 cases with filled prescriptions, all during the first or second trimester) (Table 1). Triptans were not associated with a statistically significant increased risk of prematurity (Table 1).

**Table 1:** Association between study medication exposure during pregnancy and the risk of prematurity.

Characteristics	Prematurity < 37 completed weeks of gestation			
	Yes n = 15,688	No n = 218,212	Crude RR (95% CI)	Adjusted RR (95% CI)
	n (%)			
**Study medication exposure during the pregnancy**				
DHE^a^	19 (0.12)	59 (0.03)	3.97 (1.09–14.43)	4.12 (1.21–13.99)
Triptans^b^	61 (0.39)	465 (0.21)	1.61 (1.22–2.12)	1.31 (0.99–1.74)
**Other medication use during pregnancy**				
NSAIDs	551 (3.51)	5992 (2.75)	1.18 (1.08–1.30)	1.05 (0.95–13.14)
Opioids^d^	1070 (6.82)	11,390 (5.22)	1.23 (1.15–1.32)	1.08 (1.01–1.16)
Maternal age at the 1DG^e^—mean (SD) (years)	28.12 ± 5.99	28.29 ± 5.43	1.00 (1.00–1.00)	1.00 (1.00–1.00)
Adherent vs. welfare recipient	10,922 69.62)	169,503 (77.68)	0.68 (0.65–0.70)	0.73 (0.70–0.76)
Urban dweller	12,935 (82.45)	179,671 (82.34)	1.01 (0.97–1.05)	0.97 (0.92–1.01)
Migraine diagnosis during pregnancy	446 (2.84)	4,966 (2.28)	1.22 (1.10–1.35)	1.08 (0.97–1.19)
**Maternal comorbidities in the year prior to 1DG**^**f**^				
Hypertension	667 (4.25)	5328 (2.44)	1.66 (1.53–1.81)	1.42 (1.31–1.55)
Diabetes	620 (3.95)	4688 (2.15)	1.80 (1.64–1.97)	1.52 (1.39–1.67)
Asthma	2275 (14.50)	26,046 (11.94)	1.21 (1.16–1.27)	1.04 (0.99–1.09)
Thyroid disorders	744 (4.74)	9640 (4.42)	1.07 (0.99–1.16)	0.97 (0.90–1.05)
**Diagnosis of dependence to**				
Tobacco	852 (5.43)	6531 (2.99)	1.77 (1.64–1.91)	1.52 (1.41–1.65)
Alcohol	124 (0.79)	805 (0.37)	2.04 (1.67–2.48)	1.11 (0.90–1.37)
Other drugs	372 (2.37)	2022 (0.93)	2.44 (2.17–2.74)	1.82 (1.60–2.05)
**In the year prior to the 1DG**				
Emergency visit or hospitalization	6147 (39.18)	73,409 (33.64)	1.24 (1.20–1.28)	1.07 (1.03–1.11)
**General practitioner visits**				
0	2980 (19.00)	46,466 (21.29)	Ref	Ref
1–3	5523 (35.21)	82,992 (38.03)	1.03 (0.99–1.08)	0.99 (0.95–1.04)
4 or more	7185 (45.80)	88,754 (40.67)	1.22 (1.17–1.28)	1.05 (1.00–1.10)
**Specialist visits**				
0	5,543 (35.33)	86,234 (39.52)	Ref	Ref
1–2	3,654 (23.29)	54,578 (25.01)	1.04 (0.99–1.08)	0.98 (0.93–1.02)
3 or more	6,491 (41.38)	77,400 (35.47)	1.27 (1.23–1.32)	1.08 (1.03–1.13)
**Other prescribed medications**^**g**^				
0	4257 (27.14)	69,390 (31.80)	Ref	Ref
1–2	5618 (35.81)	81,480 (37.34)	1.12 (1.08–1.17)	1.08 (1.03–1.12)
3 or more	5813 (37.05)	67,342 (30.86)	1.36 (1.31–1.42)	1.14 (1.08–1.19)
Pregnancy follow–up by obstetrician	959 (61.13	124,180 (56.91)	1.19 (1.15–1.24)	1.19 (1.15–1.23)
Pregnancy in the year prior the 1DG	1497 (9.54)	18,613 (8.53)	1.12 (1.06–1.18)	0.99 (0.93–1.05)
High dose folic acid consumption before the end of the 1st trimester	773 (4.93)	8005 (3.67)	1.33 (1.23–1.44)	1.16 (1.07–1.26)

^a^Dihydroergotamine; ^b^Including almotriptan, eletriptan, naratriptan, rizatriptan, sumatriptan, and zolmitriptan; ^c^Including celecoxib, diclofenac, diflunisal, etodolac, fenoprophen, fentanyl, flurbiprofene, hydrocodone, hydromorphone, ibuprofen, indomethacin, ketoprofen, mefenamic acid, meloxicam, nabumetone, naproxen, piroxicam, rofecoxib, sulindac, tiaprofenic acid, tolmetin, and valdecoxib; ^d^Including codeine, meperidine, morphine, oxaprozin, oxycodone, oxymorphone, pentazocine, tapentadol, propoxyphen, and tramadol.

Note: Pregnant women can use more than 1 study medication during pregnancy.

^e^First day of gestation defined as the first day of the last menstrual period; ^f^Comorbidities were assessed in the year prior to the 1DG using ICD-9 and ICD-10 diagnosis codes and prescribed medications; ^g^Other prescribed medications than the study medications and the medications used for the identification of the comorbidities.

**Table 2:** Study medication exposure during pregnancy and the risk of prematurity by sub-categories of prematurity.

Characteristics	Prematurity < 37 completed weeks of gestation n = 15,688					
	Extreme prematurity < 28 weeks of gestation		Moderate prematurity 28–31 weeks of gestation		Near-term prematurity 32–36 weeks of gestation	
	n (%)					
	Yes n = 653	No n = 233,247	Yes n = 1291	No n = 232,609	Yes	No
**Study medication exposure**						
DHE^a^	1 (0.15)	77 (0.00)	0 (0.00)	78 (0.01)	18 (0.01)	60 (0.00)
Triptans^b^	2 (0.31)	524 (0.22)	3 (0.23)	523 (0.22)	56 (0.41)	470 (0.21)

^a^Dihydroergotamine; ^b^Including almotriptan, eletriptan, naratriptan, rizatriptan, sumatriptan, and zolmitriptan.

## Low birth weight (LBW)

Within the study population, 11,875 (5.1%) pregnant women delivered a LBW newborn (Table 3). Those with a LBW newborns were older; less likely to be on welfare; more likely to be urban resident; had higher prevalence of hypertension or asthma, smoking, alcohol, and illicit drug use; more likely to be using other medications (other than the study drugs), followed by an obstetrician, and take high dose folic acid (Table 3). Adjusting for potential confounders including maternal migraine, DHE was associated with a threefold increased risk of LBW but the estimate was non-statistically significant (Table 3).

**Table 3:** Association between study medication exposure during pregnancy and the risk of LBW.

Characteristics	LBW < 2500 g at birth			
	Yes n = 11,875	No n = 222,025	Crude RR (95% CI)	Adjusted RR (95% CI)
	n (%)			
**Study medication exposure during pregnancy**				
DHE^a^	13 (0.11)	65 (0.03)	3.25 (0.76–13.92)	3.51 (0.83–14.84)
Triptans^b^	43 (0.36)	483 (0.22)	1.46 (1.06–2.02)	1.16 (0.83–1.61)
**Other medication use during pregnancy**				
NSAIDs^c^	456 (3.84)	6087 (2.74)	1.31 (1.19–1.45)	1.13 (1.03–1.25)
Opioids^d^	804 (6.77)	11,656 (5.25)	1.21 (1.12–1.31)	1.04 (0.96–1.23)
Maternal age at the 1DG^e^—mean (SD) (years)	28.16 ± 6.00	28.23 ± 5.57	1.00 (1.00–1.00)	1.00 (1.00–1.01)
Adherent vs. welfare recipient	8023 (67.56)	172,402 (77.65)	0.61 (0.59–0.64)	0.68 (0.65–0.71)
Urban dweller	9791 (82.45)	182,815 (82.34)	1.01 (0.96–1.06)	0.95 (0.90–1.00)
Migraine diagnosis during pregnancy	327 (2.75)	5085 (2.29)	1.18 (1.05–1.32)	1.05 (0.94–1.18)
**Maternal comorbidities in the year prior to 1DG**^**f**^				
Hypertension	526 (4.43)	5469 (2.46)	1.70 (1.55–1.88)	1.51 (1.37–1.67)
Diabetes	336 (2.83)	4,972 (2.24)	1.26 (1.12–1.41)	1.04 (0.93–1.17)
Asthma	1882 (15.85)	26,439 (11.91)	1.35 (1.28–1.42)	1.15 (1.09–1.22)
Thyroid disorders	541 (4.56)	9843 (4.43)	1.02 (0.94–1.12)	0.96 (0.88–1.05)
**Diagnosis of dependence to**				
Tobacco	869 (7.32)	6,514 (2.93)	2.48 (2.29–2.67)	2.09 (1.92–2.26)
Alcohol	124 (1.04)	805 (0.36)	2.77 (2.28–3.36)	1.23 (1.00–1.53)
Other drugs	348 (2.93)	2046 (0.92)	3.02 (2.68–3.41)	2.03 (1.78–2.31)
**In the year prior to the 1DG**				
Emergency visit or hospitalization	4455 (37.52)	75,101 (33.83)	1.15 (1.11–1.20)	0.99 (0.95–1.04)
**General practitioner visits**				
0	2336 (19.67)	47,110 (21.22)	Ref	Ref
1–3	4170 (35.12)	84,345 (37.99)	0.99 (0.95–1.05)	0.97 (0.92–1.03)
4 or more	5369 (45.21)	90,570 (40.79)	1.17 (1.11–1.23)	1.02 (0.97–1.08)
**Specialist visits**				
0	4370 (36.80)	87,407 (39.37)	Ref	Ref
1–2	2765 (23.28)	55,467 (24.98)	0.99 (0.95–1.04)	0.94 (0.89–0.99)
3 or more	4740 (39.92)	79,151 (35.65)	1.18 (1.13–.23)	1.01 (0.96–1.06)
**Other prescribed medications**^**g**^				
0	3271 (27.55)	70,376 (31.70)	Ref	Ref
1–2	4192 (35.30)	82,906 (37.34)	1.08 (1.04–1.14)	1.04 (1.00–1.10)
3 or more	4412 (37.15)	68,743 (30.96)	1.35 (1.29–1.41)	1.12 (1.06–1.19)
Pregnancy follow–up by obstetrician	7428 (62.55)	126,342 (56.90)	1.27 (1.22–1.32)	1.29 (1.24–1.34)
Pregnancy in the year prior the 1DG	1141 (9.61)	18,969 (8.54)	1.12 (1.05–1.20)	1.04 (0.97–1.11)
High dose folic acid consumption before the end of the 1st trimester	610 (5.14)	8168 (3.68)	1.41 (1.29–1.53)	1.27 (1.16–1.39)

^a^Dihydroergotamine; ^b^Including almotriptan, eletriptan, naratriptan, rizatriptan, sumatriptan, and zolmitriptan; ^c^Including celecoxib, diclofenac, diflunisal, etodolac, fenoprophen, fentanyl, flurbiprofene, hydrocodone, hydromorphone, ibuprofen, indomethacin, ketoprofen, mefenamic acid, meloxicam, nabumetone, naproxen, piroxicam, rofecoxib, sulindac, tiaprofenic acid, tolmetin, and valdecoxib; ^d^Including codeine, meperidine, morphine, oxaprozin, oxycodone, oxymorphone, pentazocine, tapentadol, propoxyphen, and tramadol.

Note: Pregnant women can use more than 1 study medication during pregnancy.

^e^First day of gestation defined as the first day of the last menstrual period; ^f^Comorbidities were assessed in the year prior to the 1DG using ICD-9 and ICD-10 diagnosis codes and prescribed medications; ^g^Other prescribed medications than the study medications and the medications used for the identification of the comorbidities.

## Major congenital malformations (MCM)

In this study, 24,539 newborns had an MCM (Table 4). MCM was associated with increased maternal age; hypertension, diabetes, asthma, thyroid disorders, and maternal migraine; smoking and illicit drug use; hospitalizations or emergency department visits as well as other prescribed medications (other than the studied drugs) in the year before pregnancy (Table 4). Those with an infant with MCM were also more likely to be living in urban areas, be followed by an obstetrician, and use high dose folic acid (Table 4). Adjusting for confounders including maternal migraine, gestational exposure to DHE or triptans were not associated with MCM (Table 4).

**Table 4:** Association between study medication exposure during the 1st trimester and the risk of major congenital malformations.

Characteristics	Major congenital malformation			
	Yes n = 24,539	No n = 209,361	Crude RR (95% CI)	Adjusted RR (95% CI)
	n (%)			
**Study medication exposure during the 1st trimester of pregnancy**				
DHE^a^	7 (0.03)	71 (0.03)	0.99 (0.33–2.97)	1.01 (0.32–3.19)
Triptans^b^	61 (0.25)	406 (0.19)	1.21 (0.92–1.58)	1.04 (0.79–1.37)
**Other medication use during the 1st trimester of pregnancy**				
NSAIDs	673 (2.74)	4624 (2.21)	1.20 (1.10–1.30)	1.13 (1.04–1.23)
Opioids^d^	581 (2.37)	3,904 (1.86)	1.23 (1.12–1.34)	1.14 (1.04–1.24)
Maternal age at the 1DG^e^-mean (SD) (years)	28.27 ± 5.62	28.22 ± 5.59	1.00 (1.00–1.00)	1.00 (1.00–1.00)
Adherent vs. welfare recipient	18,695 (76.18)	161,730 (77.25)	0.95 (0.92–0.98)	1.00 (0.97–1.03)
Urban dweller	20,511 (83.59)	172,095 (82.20)	1.10 (1.06–1.14)	1.08 (1.04–1.12)
Migraine diagnosis during pregnancy	674 (2.75)	4738 (2.26)	1.21 (1.12–1.32)	1.14 (1.05–1.24)
**Maternal comorbidities in the year prior to 1DG**^**f**^				
Hypertension	781 (3.18)	5214 (2.49)	1.27 (1.18–1.38)	1.14 (1.05–1.23)
Diabetes	794 (3.24)	4514 (2.16)	1.51 (1.40–1.63)	1.32 (1.22–1.43)
Asthma	3327 (13.56)	24,994 (11.94)	1.15 (1.11–1.20)	1.09 (1.04–1.13)
Thyroid disorders	1279 (5.21)	9105 (4.35)	1.21 (1.14–1.29)	1.14 (1.08–1.22)
**Diagnosis of dependence to**				
Tobacco	944 (3.85)		1.25 (1.17–1.35)	1.21 (1.12–1.30)
Alcohol	122 (0.50)		1.29 (1.06–1.56)	1.01 (0.83–1.23)
Other drugs	354 (1.44)	2040 (0.97)	1.48 (1.32–1.66)	1.35 (1.20–1.52)
**In the year prior to the 1DG**				
Emergency visit or hospitalization	9201 (37.50)	70,355 (33.60)	1.18 (1.15–1.22)	1.15 (1.12–1.19)
**General practitioner visits**				
0	4909 (20.00)	44,537 (21.27)	Ref	Ref
1–3	9184 (37.43)	79,331 (37.89)	1.05 (1.01–1.09)	1.02 (0.98–1.06)
4 or more	10,446 (42.57)	85,493 (40.84)	1.10 (1.07–1.14)	1.01 (0.96–1.15)
**Specialist visits**				
0	9229 (36.40)	82,504 (39.59)	Ref	Ref
1–2	6419 (25.32)	51,780 (24.85)	1.11 (1.07–1.15)	0.98 (0.95–1.02)
3 or more	9703 (38.27)	74,113 (35.56)	1.17 (1.13–1.20)	0.95 (0.92–0.99)
**Other prescribed medications**^**g**^				
0	7337 (29.90)	66,310 (31.67)	Ref	Ref
1–2	8842 (36.03)	78,256 (37.38)	1.02 (0.99–1.06)	1.00 (0.97–1.03)
3 or more	8360 (34.07)	64,795 (30.95)	1.16 (1.12–1.20)	1.05 (1.01–1.09)
Pregnancy follow–up by obstetrician	15,316 (62.41)	118,454 (56.58)	1.27 (1.24–1.31)	1.27 (1.23–1.31)
Pregnancy in the year prior the 1DG	2269 (9.25)	17,841 (8.52)	1.09 (1.04–1.14)	1.02 (0.97–1.07)
High dose folic acid consumption before the end of the 1st trimester	1185 (4.83)	7593 (3.63)	1.34 (1.26–1.43)	1.23 (1.15–1.31)

^a^Dihydroergotamine; ^b^Including almotriptan, eletriptan, naratriptan, rizatriptan, sumatriptan, and zolmitriptan; ^c^Including celecoxib, diclofenac, diflunisal, etodolac, fenoprophen, fentanyl, flurbiprofene, hydrocodone, hydromorphone, ibuprofen, indomethacin, ketoprofen, mefenamic acid, meloxicam, nabumetone, naproxen, piroxicam, rofecoxib, sulindac, tiaprofenic acid, tolmetin, and valdecoxib; ^d^Including codeine, meperidine, morphine, oxaprozin, oxycodone, oxymorphone, pentazocine, tapentadol, propoxyphen, and tramadol.

Note: Pregnant women can use more than 1 study medication during pregnancy.

^e^First day of gestation defined as the first day of the last menstrual period; ^f^Comorbidities were assessed in the year prior to the 1DG using ICD-9 and ICD-10 diagnosis codes and prescribed medications; ^g^Other prescribed medications than the study medications and the medications used for the identification of the comorbidities.

Table 5 presents estimates of risk of MCM by organ system. Among the 7 pregnant women exposed to DHE during pregnancy and with an infant diagnosed with MCM (Table 4), 1 had a patent ductus arteriosus and 6 had heart defects (atrial septal defects). Triptans use was associated with an increased risk of gastrointestinal defects (aRR, 2.04, 95% CI (1.01, 4.11); 9 cases with filled prescriptions); Although increases in the risk of other system defect malformations associated with the use of the study drugs during pregnancy were observed, associations were all statistically non-significant given the small number of exposed cases.

**Table 5:** Association between study medication exposures during the 1st trimester of pregnancy and the risk of organ-specific system malformations.

	**Nervous system malformations**			
	Yes	No	Crude RR	Adjusted^a^ RR
	n = 1,376	n = 232,524	(95% CI)	(95% CI)
	n (%)			
Study medication exposure during the 1st trimester of pregnancy:				
Triptans^b^	5 (0.36)	462 (0.20)	1.68 (0.70-4.02)	1.32 (0.55-3.21)
	**Eye, ear, face and neck malformations**			
	Yes	No	Crude RR	Adjusted^a^ RR
	n = 1,087	n=232,813	(95% CI)	(95% CI)
Study medication exposure during the 1st trimester of pregnancy:				
Triptans^b^	2 (0.18)	465 (0.20)	0.89 (0.22-3.58)	0.85 (0.22-3.67)
	**Patent ductus malformations**			
	Yes	No	Crude RR	Adjusted^a^ RR
	n = 962	n=232,938	(95% CI)	(95% CI)
Study medication exposure during the 1st trimester of pregnancy:				
DHE^c^	1 (0.10)	77 (0.03)	3.23 (0.39-26.69)	N.A.
Triptans^b^	3 (0.31)	464 (0.20)	1.45 (0.47-4.48)	1.06 (0.34-3.32)
	**Circulatory system malformations including heart defects**			
	Yes	No	Crude RR	Adjusted* RR
	n=5,251	n=228,649	(95% CI)	(95% CI)
Study medication exposure during the 1st trimester of pregnancy:				
DHE^c^	6 (0.11)	72 (0.03)	3.61 (0.91-14.30)	3.29 (0.89-12.14)
Triptans^b^	6 (0.11)	461 (0.20)	0.52 (0.23-1.18)	0.41 (0.18-0.93)
	**Respiratory system malformations**			
	Yes	No	Crude RR	Adjusted* RR
	n=1,777	n=232,723	(95% CI)	(95% CI)
Study medication exposure during the 1st trimester of pregnancy:				
Triptans^b^	3 (0.25)	464 (0.20)	1.15 (0.36-3.66)	0.80 (0.25-2.50)
	**Orofacial clefts malformations**			
	Yes	No	Crude RR	Adjusted^a^ RR
	n=361	n=233,539	(95% CI)	(95% CI)
Study medication exposure during the 1st trimester of pregnancy:				
DHE^c^	0	N.A.	N.A.	N.A.
Triptans^b^	0	N.A.	N.A.	N.A.
	**Gastrointestinal malformations**			
	Yes	No	Crude RR	Adjusted* RR
	n=1,713	n=232,187	(95% CI)	(95% CI)
Study medication exposure during the 1st trimester of pregnancy:				
Triptans^b^	9 (0.53)	458 (0.20)	2.43 (1.25-4.74)	2.04 (1.01-4.11)
	**Genital system malformations**			
	Yes	No	Crude RR	Adjusted* RR
	n=1,783	n=232,117	(95% CI)	(95% CI)
Study medication exposure during the 1st trimester of pregnancy:				
Triptans^b^	4 (0.22)	463 (0.20)	1.11 (0.41-3.00)	0.99 (0.37-2.69)
	**Urinary system malformations**			
	Yes	No	Crude RR	Adjusted* RR
	n=2,015	n=231,885	(95% CI)	(95% CI)
Study medication exposure during the 1st trimester of pregnancy:				
Triptans^b^	8 (0.40)	459 (0.20)	2.00 (1.00-4.03)	1.61 (0.79-3.27)
	**Musculoskeletal system malformations**			
	Yes	No	Crude RR	Adjusted* RR
	n=10,568	n=223,332	(95% CI)	(95% CI)
Study medication exposure during the 1st trimester of pregnancy:				
Triptans^b^	21 (0.20)	46 (0.20)	0.94 (0.61-1.46)	0.86 (0.55-1.34)

^a^Adjusted for all variables included in the previous table.

^b^Including almotriptan, eletriptan, naratriptan, rizatriptan, sumatriptan, and zolmitriptan; ^c^Dihydroergotamine.

Pregnant women can use more than 1 study medication during pregnancy.

## Spontaneous abortions (SA)

Twenty-nine thousand one hundred and four (29,104) cases of SA were identified, and 287,607 matched controls were analyzed (Fig. 2, Table 6). Women with a SA were older, less likely to be on welfare, and living in urban areas; more likely to have a history of migraine, hypertension, and asthma; smoked and drank alcohol, and used illicit drugs more than those who did not miscarry; had more hospital and physician visits in the year before pregnancy; were less likely to be followed by obstetrician and take high dose folic acid (Table 6). They were also more likely to have had another pregnancy in the 12 months before the index pregnancy. Adjusting for potential confounders including maternal migraine, use of triptans during pregnancy was associated with the risk of SA (aOR, 1.63, 95% CI (1.34, 1.98); 192 cases with filled prescriptions); DHE was associated with a doubling of the risk of SA but the estimate was non-statistically significant (Table 6).

**Table 6:** Association between study medication exposure during pregnancy and the risk of spontaneous abortion.

	Spontaneous abortion			
Characteristics	Cases n = 29,104	Controls n = 287,607	Crude OR (95% CI)	Adjusted OR (95% CI)
	n (%)			
**Study medication exposure during the pregnancy**				
DHE^a^	22 (0.08)	64 (0.02)	3.79 (1.27–11.34)	2.59 (0.79–8.53)
Triptans^b^	192 (0.66)	713 (0.25)	1.99 (1.67–2.38)	1.63 (1.34–1.98)
**Other medication exposure during pregnancy**				
NSAIDs^c^	2423 (8.33)	8054 (2.80)	2.70 (2.56–2.85)	2.60 (2.46–2.76)
Opioids^d^	1529 (5.25)	7433 (2.58)	1.36 (1.27–1.45)	1.53 (1.43–1.64)
**Maternal age at the 1DG**^**e**^**—years**				
Less than 35	22,253 (76.46)	241,761 (83.96)	Ref	Ref
35–39	4711 (16.19)	37,291 (12.95)	1.36 (1.32–1.41)	1.69 (1.63–1.75)
40 or more	2140 (7.35)	8,884 (3.09)	2.60 (2.46–2.74)	3.24 (3.06–3.43)
Adherent vs. welfare recipient	20,730 (71.23)	214,977 (74.66)	0.85 (0.82–0.87)	1.02 (0.99–1.06)
Urban dweller	22,910 (78.72)	231,234 (80.31)	0.90 (0.87–0.93)	0.97 (0.94–1.01)
Migraine diagnosis during pregnancy	622 (2.14)	5112 (1.78)	1.20 (1.10–1.31)	0.98 (0.89–1.08)
**Maternal comorbidities in the year prior to 1DG**^**f**^				
Hypertension	929 (3.19)	7820 (2.72)	1.20 (1.11–1.29)	1.06 (0.98–1.15)
Diabetes	728 (2.50)	8495 (2.95)	0.75 (0.69–0.82)	0.80 (0.74–0.88)
Asthma	4375 (15.03)	36,307 (12.61)	1.19 (1.15–1.23)	1.00 (0.96–1.04)
Thyroid disorders	1322 (4.54)	15,584 (5.41)	0.78 (0.73–0.83)	0.75 (0.71–0.80)
**Diagnosis of dependence to**				
Tobacco	445 (1.53)	2829 (0.98)	1.56 (1.40–1.74)	1.08 (0.96–1.22)
Alcohol	228 (0.78)	1035 (0.36)	2.09 (1.79–2.44)	1.31 (1.10–1.57)
Other drugs	381 (1.31)	2035 (0.71)	1.83 (1.63–2.05)	1.21 (1.06–1.38)
**In the year prior to the 1DG**				
Emergency visit or hospitalization	12,059 (41.43)	105,223 (36.54)	1.21 (1.18–1.24)	0.94 (0.91–0.97)
**General practitioner visits**				
0	5217 (17.93)	62,341 (21.65)	Ref	Ref
1–3	9808 (33.70)	108,303 (37.61)	1.08 (1.04–1.12)	1.04 (1.00–1.08)
4 or more	14,079 (48.37)	117,292 (40.74)	1.43 (1.38–1.48)	1.12 (1.07–1.17)
**Specialist visits**				
0	10,544 (36.23)	112,271 (38.99)	Ref	Ref
1–2	6828 (23.46)	70,740 (24.57)	1.03 (1.00–1.07)	1.12 (1.08–1.17)
3 or more	11,732 (40.31)	104,925 (36.44)	1.18 (1.15–1.21)	1.38 (1.33–1.43)
**Other prescribed medications**^**g**^				
0	8144 (27.98)	91,446 (31.76)	Ref	Ref
1–2	9960 (34.22)	105,108 (36.53)	1.06 (1.03–1.09)	1.02 (0.98–1.05)
3 or more	11,000 (37.80)	91,310 (31.71)	1.34 (1.30–1.39)	1.19 (1.15–1.24)
Pregnancy follow–up by obstetrician	3332 (11.45)	121,836 (42.31)	0.17 (0.16–0.18)	0.15 (0.14–0.16)
Pregnancy in the year prior the 1DG	5386 (18.51)	42,971 (14.92)	1.26 (1.22–1.31)	1.10 (1.06–1.15)
High dose folic acid consumption before the end of the 1st trimester	885 (3.04)	12,691 (4.41)	0.69 (0.64–0.74)	0.69 (0.64–0.75)

^a^Dihydroergotamine; ^b^Including almotriptan, eletriptan, naratriptan, rizatriptan, sumatriptan, and zolmitriptan; ^c^Including celecoxib, diclofenac, diflunisal, etodolac, fenoprophen, fentanyl, flurbiprofene, hydrocodone, hydromorphone, ibuprofen, indomethacin, ketoprofen, mefenamic acid, meloxicam, nabumetone, naproxen, piroxicam, rofecoxib, sulindac, tiaprofenic acid, tolmetin, and valdecoxib; ^d^Including codeine, meperidine, morphine, oxaprozin, oxycodone, oxymorphone, pentazocine, tapentadol, propoxyphen, and tramadol.

Note: Pregnant women can use more than 1 study medication during pregnancy.

^e^First day of gestation defined as the first day of the last menstrual period; ^f^Comorbidities were assessed in the year prior to the 1DG using ICD-9 and ICD-10 diagnosis codes and prescribed medications; ^g^Other prescribed medications than the study medications and the medications used for the identification of the comorbidities.

## Additional analyses

Sensitivity analyses on DHE categorization of duration of exposure before and during pregnancy gave results similar to the primary analyses (Tables 7, 8, 9, and 10). More specifically those using DHE before and during pregnancy were at increased risk of prematurity (aRR, 2.59, 95% CI (1.17, 5.73); 13 cases with filled prescriptions); those using DHE during pregnancy but not before pregnancy were at increased risk of prematurity but the estimate was not statistically significant (aRR, 1.26, 95% CI (0.69, 2.30); 6 cases with filled prescriptions) (Table 7). DHE exposure was mostly during the first trimester up to the 15^th^ week of pregnancy among those who used it during gestation only whereas DHE was discontinued before the 8^th^ week of pregnancy among those who used it before and during gestation.

**Table 7:** Association between DHE exposure before or during pregnancy and the risk of prematurity by categories of DHE exposure.

Characteristics	Prematurity < 37 completed weeks of gestation			
	Yes n = 15,688	No n = 218,212	Crude RR (95% CI)	Adjusted RR (95% CI)
	n (%)			
**Study medication exposure during the pregnancy**				
DHE^a^ prior to pregnancy only	8 (0.05)	83 (0.04)	1.19 (0.78–1.82)	1.12 (0.73–1.72)
DHE prior and during pregnancy	13 (0.08)	65 (0.03)	2.65 (1.11–6.33)	2.59 (1.17–5.73)
DHE during pregnancy only	6 (0.04)	72 (0.03)	1.30 (0.79–2.14)	1.26 (0.69–2.30)

Adjusted for all variables included in Table 1.

^a^Dihydroergotamine.

**Table 8:** Association between DHE exposure before or during pregnancy and the risk of LBW by categories of DHE exposure.

Characteristics	LBW < 2500 g at birth			
	Yes n = 11,875	No n = 222,025	Crude RR (95% CI)	Adjusted RR (95% CI)
	n (%)			
**Study medication exposure during pregnancy**				
DHE^a^ prior to pregnancy only	7 (0.06)	84 (0.04)	1.45 (0.71–2.96)	1.41 (0.73–2.72)
DHE prior and during pregnancy	8 (0.07)	70 (0.03)	2.30 (0.76–6.96)	2.21 (0.81–6.03)
DHE during pregnancy only	5 (0.04)	73 (0.03)	1.33 (0.66–2.68)	1.25 (0.68–2.30)

Adjusted for all variables included in Table 3.

^a^Dihydroergotamine.

**Table 9:** Association between DHE exposure before or during the 1st trimester and the risk of major congenital malformations by categories of DHE exposure.

Characteristics	Major congenital malformation			
	Yes n = 24,539	No n = 209,361	Crude RR (95% CI)	Adjusted RR (95% CI)
	n (%)			
**Study medication exposure during the 1st trimester of pregnancy**				
DHE^a^ prior to pregnancy only	7 (0.03)	84 (0.04)	0.79 (0.33–1.89)	0.84 (0.35–2.02)
DHE prior and during pregnancy	6 (0.02)	72 (0.03)	0.69 (0.28–1.70)	0.71 (0.30–1.68)
DHE during pregnancy only	1 (0.004)	77 (0.04)	N.A	N.A

Adjusted for all variables included in Table 4.

^a^Dihydroergotamine.

**Table 10:** Association between DHE exposure before or during pregnancy and the risk of spontaneous abortion by categories of DHE exposure.

Characteristics	Spontaneous abortion			
	Cases n = 29,104	Controls n = 287,607	Crude OR (95% CI)	Adjusted OR (95% CI)
	**n (%)**			
**Study medication exposure during the pregnancy**				
DHE^a^ prior to pregnancy only	12 (0.04)	83 (0.03)	1.28 (0.91–1.80)	1.27 (0.89–1.81)
DHE prior and during pregnancy	18 (0.06)	68 (0.02)	3.12 (1.01–9.64)	2.71 (0.81–9.07)
DHE during pregnancy only	4 (0.01)	82 (0.03)	0.39 (0.11–1.38)	0.46 (0.13–1.63)

Adjusted for all variables included in Table 6.

^a^Dihydroergotamine.

Finally, the E-value obtained for the association between DHE and prematurity was 4.3 with a lower limit of 1.02; DHE and LBW was 3.32 with a lower limit of 0.89; DHE and MCM was 0.99 with a lower limit of 0.29; and DHE and SA was 2.61 with a lower limit of 0.81. The E-value obtained for the association between triptan and prematurity was 1.57 with a lower limit of 0.94; triptan and LBW was 1.27 with a lower limit of 0.84; triptan and MCM was 1.01 with a lower limit of 0.79; and triptan and SA was 1.74 with a lower limit of 1.41. These are all suggesting that unmeasured confounding was unlikely to explain the findings.

Table 11 summarizes study findings with regards to statistically significant increased risks.

**Table 11:** Summary of statistically significant study findings.

Study medications	Prematurity	LBW	MCM	Spontaneous abortion
DHE^a^	X			
Triptans^b^				X

^a^Dihydroergotamine; ^b^Including almotriptan, eletriptan, naratriptan, rizatriptan, sumatriptan, and zolmitriptan.

## Discussion

In this population-based pregnancy cohort, both DHE and triptans used for the acute treatment of migraine were associated with some adverse pregnancy outcomes. We found that DHE was associated with the risk of prematurity, and triptans were associated with an increased risk of SA.

Although the study cohort was large, the prevalence of DHE and triptan use was low, and some estimates are unstable and lack statistical power. The study has shown that DHE gestational exposure increases the risk of prematurity, and gestational exposure to triptans is associated with an increased risk of SA, even after taking into account the risk attributed to maternal migraine.

Our findings on DHE are similar to other studies. The risk of congenital malformations in relation to the vasoconstrictive effects of ergotamine has been assessed within a population-based cohort in Hungary^19^. They did not find an association between ergotamine and the risk of congenital malformations (OR 1.3, 95% CI 0.6–2.7); our finding was close to a null effect. A difference between their study and ours is however, that they looked at exposure to ergotamine, which is teratogenic. Additionally, in animal studies, hydrogenated ergot alkaloids such as DHE have been shown to reduce uterine stimulating properties relative to the parent ergot alkaloids such as ergotamine^20^. Another difference is that our study did not find a statistically significant association for LBW, although this finding might lack statistical power. Nevertheless, both studies found an increased risk of prematurity.

Our findings on LBW and malformations are also comparable to those from Kallen et al.^21^ which utilized data from the Swedish Medical Birth Register. Kallen et al.^21^ did not find an increased risk of prematurity with gestational exposure to DHE, which could be explained by different timing of exposures during pregnancy. Indeed, although we do not have the exact timing of migraine drug exposures in Kallen et al.^21^, DHE exposures were mostly during the first half of pregnancy in our study. DHE can induce uterine contractions, which can result in preterm delivery^22^.

Our results on triptans are consistent with the literature. Indeed, we did not find that triptan use during pregnancy was increasing the risk of MCM similarly as in other studies from Norway and Sweden^21,23^. Our finding on SA is also consistent with a recent meta-analysis that suggested an increased risk but lacked statistical power given that only two studies were included^15^. Our findings on prematurity and LBW need to be replicated in other studies.

This large population-based study enabled us to evaluate the effect of acute-treatment-of-migraine drug use during pregnancy. Data on filled prescriptions have been validated^24^, and do not rely on maternal recall, which could be prone to recall bias if assessed retrospectively, and thus exposure misclassification^25^. Prescription fillings, filled prospectively as part of usual care, as is the case here, are better measures of medication exposures during pregnancy, especially when exposure is dichotomized as was done in this study. Moreover, Jonge et al.^26^ have shown that pregnant women filling prescriptions had 84–92% likelihood of taking at least one dose, enhancing the validity of data. Nevertheless, it remains that maternal reports can be better than prescription fillings when they are collected in real-time prospectively, especially for medications that are taken on an as-needed basis. Diagnoses of major malformations^27^ as well as data on birth weight^28^ have been validated against patient charts. Gestational age was also validated and obtained in patients charts at index date, which enabled us to calculate timing of DHE, and triptan exposure during pregnancy^28^. Although we have not censored the time-window of exposure for our analyses on prematurity, all DHE and triptan exposures occurred before the 21^st^ week of gestation, which did not impact our estimates of risks. Furthermore, given that all study medication exposures occurred during organogenesis or shortly after, our findings on MCM are biologically plausible. Only clinically detected spontaneous abortions were considered, without relying on maternal recall. Spontaneous abortions that were never detected by the women themselves were excluded, as was done in all other similar studies to date^29,30^. If DHE or triptans increase the risk of spontaneous abortions that are not clinically detected, our findings are conservative and thus would underestimate the true risk. However, if they are not associated with non-clinically detected spontaneous abortions, there is no reason to believe that misclassification would be different between cases and controls, resulting in non-differential misclassification. We adjusted our findings for maternal migraine, hence limiting potential confounding by indication. We also studied all specific and non-specific acute migraine therapeutics within a single population-based pregnancy cohort, which considered migraine severity, and use of concomitant or complementary therapeutics during gestation. Although restricting our study cohort to only those with migraine would have been another option for adjusting for the indication, it would not have allowed us to compare the risk of prematurity, LBW, MCM and SA with that for non-specific migraine medications. In addition, given the data that we have (billing, hospital data) are for 1 year before pregnancy and during pregnancy, a woman could have had a diagnosis of migraine once and never have the diagnosis again in the year before and during pregnancy, while being on anti-migraine treatment. Hence, we know that if she uses a specific anti-migraine medication (triptan or DHE), she is using it for migraine. Given that there could be misclassification on migraine diagnosis, we have done sensitivity analysis, further categorizing DHE use, which gave similar results. Therefore, we are confident that residual confounding by indication, if present, would not completely explain our findings. The evaluation of exposure, although validated, was based on filled prescriptions and might not necessarily reflect actual intake. However, we hypothesize that women who filled a prescription for a DHE or triptan took at least one dose, since there is a co-pay. The fact that anti-migraine medications are used on a as needed basis is a further justification for dichotomizing exposure during pregnancy. Furthermore, de Jonge et al.^26^ have shown that during pregnancy, the compliance rates of medication fillings ranged from 0.84 (for chronic diseases) to 0.92 (for pregnancy-related symptoms); most of the medications actually taken were used at the prescribed dosage or lower; and more than half of the medications actually taken were used for the duration prescribed or shorter. Nevertheless, no data on dosage levels or timing of use during an episode of migraine were available in our study, which remains a limitation. Finally, the four analyses permitted us to select subjects originating from the same source population—the QPC—limiting the potential for selection bias.

Within the QPC, information on potential confounders such as maternal obesity and over-the-counter (OTC) folic acid use are not available. Nevertheless, we have shown that maternal weight and pre-conceptual OTC folic acid intake are not strong enough confounding variables to overturn findings of associations between medication exposure during gestation and adverse pregnancy outcomes^31^. Maternal weight has been shown to be associated with the risk of MCM^32^; however, it is unlikely that women using DHE or triptan would differ significantly in their weight, and thus this would not explain the differences in the risk estimations. The sensitivity analyses performed with E-values are reassuring, although unmeasured confounding could still explain some of our findings. We have taken into account prescribed folic acid use, hence prescribed OTC use, and high dose use, which requires a prescription. Given that high dose folic acid is given to high-risk pregnancies, our estimates are likely markers of severity. Indeed, high dose folic acid is indicated for women with previous pregnancies resulting in children with neural tube defects or any other malformations, women with risky lifestyles (cigarette smoking, illicit drug use), diabetic women, etc^33^. Although we have adjusted for these (in part), it remains that users of high dose folic acid are women with ‘at-risk’ pregnancies, i.e. pregnancies that are at increased risk of adverse perinatal outcomes, which could explain our findings. Finally, in Quebec, only ibuprofen NSAID is available OTC, which is less problematic than in other countries where ibuprofen and naproxen are available OTC. Nevertheless, we do have data on filled prescribed OTC medications. Although it can potentially misclassify exposure in our analyses on NSAIDs, our study subjects come from a lower socio-economic stratum, which increases the chance of them having a prescribed OTC. Nevertheless, misclassification of NSAIDs exposure is possible, which would increase the likelihood of having pregnant NSAID users in our defined non-user group. This would underestimate the true risk. Therefore, our estimates for NSAIDs are potentially underestimated. Similarly, acetaminophen OTC is not well captured in the QPC. We have considered acetaminophen exposure in our covariate ‘other prescribed medications’ in our models, but we cannot rule out exposure misclassification for this variable. In our study, migraine was not associated with an increased risk of adverse pregnancy outcomes, and we have adjusted for history of migraines, and use of health care services, non-specific anti-migraine medication use, and other maternal comorbidities, which could all be proxies for migraine severity; still, we cannot completely rule out the possibility of residual confounding by underlying disease in the risk estimates for DHE or triptans. The MCM prevalence of 10% is higher than what is routinely reported (3–5%)^34^. This is a known fact in the province of Quebec referred to as the Founder’s effect^35–38^. Although our baseline prevalence of MCMs is high, it does not differ among our compared groups, and therefore does not invalidate our findings. This has been mentioned before in studies emerging from Quebec^38^. The prevalence of SA is also high in the QPC, which is in concordance with population reported values^37^. Furthermore, we cannot exclude the possibility of chance findings in 5% of our statistically significant associations. For the analyses of specific adverse pregnancy outcomes, significant associations might have been missed due to lack of statistical power, and estimates could be unstable given the small number of pregnant women taking DHE, or a triptan. More specifically, the wide confidence interval for the association between DHE use during pregnancy and the risk of prematurity means the estimate is unstable and that the data may be consistent with a wide range of other hypotheses. Although GEE models were used to analyze our data, Cox proportional models have also been used in other pregnancy studies, and would have been appropriate options^39,40^. A Cox proportional model would be useful when drug exposure fillings are analyzed using the number of days exposed, to take into account the time varying exposure, which is not the case in our study. Indeed, we have dichotomized drug exposure (yes vs. no), given that DHE and tryptans are used as needed, and not necessarily as prescribed or filled. Furthermore, the majority of DHE and tryptan filled prescriptions occurred in the first trimester, which is relevant because a similar time-window of exposure was present between those with preterm and term births (in our study, data show that the exposure time-window does not change depending on prematurity status). In this present situation, both models would lead to similar findings.

Lastly, given that we only studied pregnant women insured by the Quebec public drug insurance program, generalizability can be affected. Nevertheless, Bérard and Lacasse^41^ have shown that although pregnant women insured by private vs. public medication insurance plans differed with regards to socio-economic status, they are similar in terms of comorbidities. In addition, the Quebec Pregnancy Cohort characteristics are similar to what has been reported in provincial and national health surveys, which is reassuring (Bérard and Sheehy)^37^.

## Conclusions

Even after considering maternal migraine and concomitant migraine medication use, all antimigraine medications increased the risk of some adverse pregnancy outcomes. This study showed that, other than for prematurity, the risk of DHE use during pregnancy was similar to that of triptan use. Although this updated enhanced analysis gave similar conclusions with regards to DHE and the risk of prematurity as was shown in Bérard and Kori^8^, it is based on 6 more years of data, more exposed cases, and increased statistical power. Furthermore, we have shown that our results are unlikely due to indication bias or unmeasured confounders. It remains however, that further confirmation in a larger cohort is warranted. Given that a recent study showed that almost 9 out of 10 women reported deliberate non-adherence to needed antimigraine medications during pregnancy out of fear of harming their unborn children^42^, there is need for more publicly available and consistent information regarding the potential risks of antimigraine medication use during pregnancy.

## Methods

### Study cohort

We conducted a population-based cohort study using data from the Quebec Pregnancy Cohort (QPC), built with the linkage of three databases in Quebec. The QPC is an ongoing population-based cohort with prospective data collection on all pregnancies that occurred between January 1998 and December 31, 2015 in the province of Quebec. Data on the mothers and children were also collected after birth until December 2015. Individual-level information was obtained from province-wide databases and linked using unique personal identifiers. The QPC was first constructed by identifying all pregnancies in the Régie de l’assurance maladie du Québec (RAMQ) and the Quebec hospitalization archives (MedEcho) databases; subsequently, the first day of the last menstrual period (first day of gestation: 1DG) was defined using data on gestational age, which was validated against patients’ charts^28^. Prospective follow-up was available from 1 year before the 1DG, during pregnancy, and until December 31, 2015.

Analyses of spontaneous abortions (SA) were based on all pregnancies in the cohort, whereas analyses of major congenital malformations (MCM), prematurity and LBW were based on singleton livebirths. This was done because multiple births status is an effect modifier in analyses on MCM, prematurity, and LBW. Analyses on SA considered all pregnancies, given that no data on multiplicity is available at the time of the event in the QPC, and these analyses only consider events that are clinically detected (excluding SA that occur before women would realize they are pregnant). The QPC data sources for this study included the medical service database (RAMQ: diagnoses, medical procedures, socio-economic status of women and prescribers), the Quebec’s Public Prescription Drug Insurance database (drug name, start date, dosage, duration), the hospitalization archive database (MedEcho: diagnoses and procedures), and the Quebec Statistics database (ISQ: patient socio-demographic, birth weight). The QPC is further described in Bérard and Sheehy^37^.

We included pregnancies with continuous Prescription Drug Insurance coverage of at least 12 months before the 1DG and during pregnancy; all pregnancies meeting this criterion were considered and analyzed. We excluded pregnancies exposed to known teratogens during the 1st trimester of pregnancy (0–14 completed weeks of gestation) as described by Kulaga et al.^43^ For the analyses of SA, we excluded women with planned abortions or women whose abortions occurred at a gestational age of less than 6 completed weeks of gestation (these are potentially subjected to misclassification because many early pregnancy losses are not recognized clinically). We also excluded pregnancies with SA after 22 weeks of gestation, which is clinically implausible. Furthermore, for analyses of MCM, we excluded pregnancies resulting in minor malformations alone or in chromosomal abnormalities in newborns. This was done because minor malformations are probably diagnosed selectively (hence detection bias, misclassification of the outcome), and chromosomal abnormalities are unlikely to be due to medication exposures.

The study was approved by the Quebec Data Access Agency and the CHU Sainte-Justine Institutional Review Board.

### Study design

Cohort design.

### Exposure to DHE and triptans

Migraine-specific medications were considered as study medications; all routes of administrations were considered (oral, injection, nasal spray, suppository). Exposure was defined as having filled at least one prescription for DHE, or triptan (almotriptan, eletriptan, naratriptan, rizatriptan, sumatriptan, and zolmitriptan) during pregnancy. Frovatriptan is not on the list of reimbursed medications in Quebec. Hence, we do not have data on this molecule, and it was not included in the study. Prescription fillings before pregnancy with durations overlapping with the beginning of gestation were also defined as a pregnancy exposure. When studying SA, the exposure period of interest was defined as the beginning of pregnancy until the date of the SA or the corresponding index date for the matched comparators; for prematurity, LBW, or MCM, the exposure period of interest was any time during pregnancy. Given that anti-migraine medications are used on a as needed basis, exposure status was defined dichotomously during the exposure time period of interest.

Data on prescription fillings in the QPC have been validated and compared to maternal reports, which is more reliable than data on medication prescribing in medical charts; the positive predictive value of prescription drug data in the cohort was found to be at least 87% (95% CI: 70%-100%) and the negative predictive value was at least 92% (95% CI: 86%-98%)^24^. In addition, a co-payment is required for all fillings, increasing the likelihood of medication intake.

### Outcomes

Cases of MCM diagnosed in the first year of life were identified in the QPC with data from the RAMQ and MedEcho databases and defined according to ICD-9 codes (740–759 excluding codes of minor congenital malformations or chromosomal abnormalities: 743.6, 744.1–744.4, 744.8, 744.9, 747.0, 747.5, 750.0, 752.4, 752.5, 754.6, 755.0, 755.1, 757.2–757.6, 757.8, 757.9, 758.4) and ICD-10 codes (Q00-Q99, excluding codes of minor malformations or chromosomal abnormalities: Q08-Q10, Q162, Q17-Q19, Q250, Q270, Q381, Q515, Q516, Q20-Q53, Q664-Q666, Q689, Q70, Q81-Q84, Q94-Q95). ICD-9 and ICD-10 codes of major congenital malformations in the QPC have been validated against patient charts^27^. The positive predictive value of major congenital malformations diagnosed in the first year of life in the QPC have been found to be at least 80% and the negative predictive value 93%^27^. All organ systems were considered.

Cases of prematurity were identified in the QPC with the hospital archives database (MedEcho: gestational age) and defined as a delivery at < 37 completed weeks’ gestation.

Cases of LBW were identified in the QPC with the Quebec Statistics database (ISQ: birth weight) and defined as a newborn weighting < 2500 g. Gestational age and birth weight have been validated against patients’ charts^28^.

Cases of spontaneous abortions were identified in the QPC using the RAMQ database with ICD-9–10 codes 630–634, 690.0 and 690.9. Only cases diagnosed between the 6th and 22nd weeks of gestation were included. Planned abortions were excluded from analyses.

### Statistical analyses

Four separate analyses were performed within the study cohort. MCM, prematurity and LBW were defined at delivery using the previously mentioned definitions, and using a traditional closed cohort design^44^.

For the analyses of spontaneous abortions, we performed a nested case–control design within the study cohort. Cases were defined as women with a diagnosis or a procedure for spontaneous abortion between the 6th and the 22nd week of gestation. The index date was defined as the calendar date of the spontaneous abortion. Because of our plan to assess several specific anti-migraine medications simultaneously, we randomly selected up to 10 controls for each case matched on gestational age and calendar year at the time of the event (spontaneous abortion or matched index date for selected controls). Similar to the methods of Einarson and colleagues^45^, we matched controls by the case’s index date and thus gestational age at the time of the spontaneous abortion. We did this because the risk of pregnancies ending in a loss is highly dependent on the gestational age at which the pregnancy is recognized, and because the probability of a spontaneous abortion being clinically detected increases with gestational age. Therefore, using a nested case–control design, we selected controls from among women included in the Quebec Pregnancy Cohort who did not have a spontaneous abortion at or before the same gestational age as their matched case did. The index date of the controls was the same as that for the matched case. The nested case–control design gives similar effect sizes as the prospective cohort approach but has greater computational efficiency^44,46^. Furthermore, this design for the study of spontaneous abortions has been used before in pregnancy studies^29,45,47^.

Potential confounders considered for all analyses were known risk factors or associated with risk factors for the 4 studied outcomes (all these variables were either risk factors or determinants for adverse pregnancy outcomes): (1) sociodemographic variables on the 1DG including maternal age, welfare status during pregnancy or 1 year before (yes/no), and area of residence (urban/rural); (2) Maternal chronic co-morbidities during the 12 months prior to pregnancy including hypertension, diabetes (Type I or II), asthma, and thyroid disorders. The previous conditions were identified from either diagnoses or filled prescriptions of related medications; (3) smoking, alcohol of illicit drug use before pregnancy; (4) Health care utilization during the 12 months prior to the 1DG until the end of the critical time-window for the outcome studied including hospitalizations or emergency department (ED) visits (yes/no), visits to a specialist or general practitioner (yes/no); (5) diagnosis of migraine (yes/no) in the year before 1DG and during pregnancy to take into account indication bias; (6) Pregnancy related variables including previous pregnancy in the year prior to the 1DG (yes/no). We also took into account whether pregnant women were followed by an obstetrician (yes/no), and if other medications, including non-steroidal anti-inflammatory drugs (NSAIDs) or opioids, were used during pregnancy. Finally, use of high dose folic acid (> 5 mg/d) or prescribed low dose folic acid before or during pregnancy was measured. Univariate and multivariate generalized estimation equation (GEE) models with the genmod function were built to quantify the independent association between the use of DHE, or triptans during pregnancy and the risk of prematurity, LBW, major congenital malformations, and spontaneous abortions adjusting for clinically important confounders and socioeconomic variables. The GEE models were also used to take into account inter-pregnancy variations as well as within-woman variations for those with multiple pregnancies between 1998 and 2015. Furthermore, the great advantage of GEE is that it provides parameter estimates and their (asymptotically) correct standard errors, and hence (asymptotically) correct inferences (tests, confidence intervals, etc.) even in cases when the correlation structure is not correctly specified^48^. It can be used for cohort and case–control studies as well as nested-case–control designs. Both DHE and tryptan prescribed fillings were considered in the univariate models on anti-migraines to take into account concomitant use during pregnancy.

Given that ergotamine has been shown to be teratogenic in animal and human studies, we wanted to further analyze DHE by stratifying on duration of DHE exposure before and during pregnancy. Sensitivity analyses were performed stratifying DHE exposure to further take into account indication bias as follows: exposed to DHE (i) in the 12 months prior to pregnancy but not during pregnancy, (ii) in the 12 months prior to pregnancy and during pregnancy, and (iii) during pregnancy but not in the 12 months prior to pregnancy. The hypothesis is that if pregnant women used DHE before pregnancy but paused during pregnancy, such estimate should be null in the absence of indication bias. However, if an increased risk of prematurity is seen in this group, it is likely the result of the indication and not the medication. This analysis has been used before in another study^49^.

We also calculated E-values to measure the robustness of the association between DHE and triptan use and prematurity, LBW, MCM, and SA for unmeasured or unadjusted confounding using the new measure proposed by VanderWeele and Ding^50^.

Risk ratios (RR; prematurity, LBW, MCM) and odds-ratios (OR; SA) with 95% confidence intervals (CI) were calculated using SAS System for Windows (SAS Institute Inc, North Carolina, USA). Differences were considered statistically significant when the 95% CIs did not overlap 1.0 and when P values (2-tailed) were less than 0.05.

### Ethics approval and consent to participate

Ethical approval for this study was obtained from CHU Sainte-Justine Institutional Review Board [approval number 1740 and 2976]. The Quebec “Commission d’Accès à l’information” authorized database linkages.

### Informed consent

Informed consent was not sought for the present study because using linked administrative health records databases, we only have access to anonymous (person non-identifiable) data.

## Data Availability

The data supporting the findings of this study are available from the corresponding author upon reasonable request.
